# Remote patient monitoring for COVID-19 patients: comparisons and framework for reporting

**DOI:** 10.1186/s12913-023-09526-0

**Published:** 2023-08-03

**Authors:** David Joyce, Aoife De Brún, Sophie Mulcahy Symmons, Robert Fox, Eilish McAuliffe

**Affiliations:** Interdisciplinary Research Education and Innovation in Health Systems (IRIS) Centre, School of Nursing, Midwifery and Health Systems, University College Dublin, Dublin, D04 V1W8 Ireland

**Keywords:** Remote patient monitoring, COVID-19, Reporting guidelines, Framework, Implementation, Technology

## Abstract

**Background:**

COVID-19 has challenged health services throughout the world in terms of hospital capacity and put staff and vulnerable populations at risk of infection. In the face of these challenges, many health providers have implemented remote patient monitoring (RPM) of COVID-19 patients in their own homes. However systematic reviews of the literature on these implementations have revealed wide variations in how RPM is implemented; along with variations in particulars of RPM reported on, making comparison and evaluation difficult. A review of reported items is warranted to develop a framework of key items to enhance reporting consistency.

The aims of this review of remote monitoring for COVID-19 patients are twofold:

(1) to facilitate comparison between RPM implementations by tabulating information and values under common domains.

(2) to develop a reporting framework to enhance reporting consistency.

**Method:**

A review of the literature for RPM for COVID-19 patients was conducted following PRISMA guidelines. The Medline database was searched for articles published between 2020 to February 2023 and studies reporting on items with sufficient detail to compare one with another were included. Relevant data was extracted and synthesized by the lead author. Quality appraisal was not conducted as the the articles considered were evaluated as informational reports of clinical implementations rather than as studies designed to answer a research question.

**Results:**

From 305 studies retrieved, 23 studies were included in the review: fourteen from the US, two from the UK and one each from Africa, Ireland, China, the Netherlands, Belgium, Australia and Italy. Sixteen generally reported items were identified, shown with the percentage of studies reporting in brackets: Reporting Period (82%), Rationale (100%), Patients (100%), Medical Team (91%) Provider / Infrastructure (91%), Communications Platform (100%), Patient Equipment (100%), Training (48%), Markers (96%), Frequency of prompt / Input (96%),Thresholds (82%), Discharge (61%), Enrolled (96%), Alerts/Escalated (78%), Patient acceptance (43%), and Patient Adherence (52%).

Whilst some studies reported on patient training and acceptance, just one reported on staff training and none on staff acceptance.

**Conclusions:**

Variations in reported items were found. Pending the establishment of a robust set of reporting guidelines, we propose a reporting framework consisting of eighteen reporting items under the following four domains: Context, Technology, Process and Metrics.

**Supplementary Information:**

The online version contains supplementary material available at 10.1186/s12913-023-09526-0.

## Background

COVID-19 has challenged health services throughout the world in terms of hospital capacity [1, 2], whilst nosocomial transmission continues to put staff and vulnerable populations at risk of infection [3, 4].

In the face of these challenges, the role of telemedicine has received new impetus. A systematic review on the role of telehealth during the early COVID-19 outbreak concludes that the use of telehealth improves the provision of health services and, therefore, telehealth should be an important tool in caring services while keeping patients and health providers safe during COVID-19 [5]. One particular aspect of telehealth, remote monitoring of patients in their own homes, was considered a potential solution to avoid overburdening hospital capacity and mitigating the risk of nosocomial infection. The US based Food and Drugs Administration (FDA) responded early by issuing a policy in March 2020 to facilitate greater use of RPM technologies to reduce hospital visits [6].

RPM has been implemented for COVID-19 patients by various health services. Of necessity, many of the reported implementations of RPM have been introduced independently and at speed, without the opportunity to learn one from another. A study on how health systems learn from one another suggests that learning from wider contexts is critical in order to improve performance [7].

A systematic review of 27 studies [8] on RPM for COVID-19 sought to determine the impact of remote home monitoring on virtual length of stay, escalation, emergency department attendance/reattendance, admission/readmission and mortality. It was able to determine that most implementations were led by secondary care, that a positive test for COVID-19 was not required in most cases for patient eligibility and that monitoring was conducted via online platforms, paper-based systems with telephone calls or (less frequently) through wearable sensors. However, the review clearly states that it was difficult to carry out an analysis of the impact of remote home monitoring across all examples because not all articles reported data on the same outcomes and that it could not reach substantive conclusions regarding patient safety and the identification of early deterioration due to lack of standardised reporting and missing data.

Guidelines that specify a minimum set of criteria for reporting can improve the accuracy and transparency of publications, thus facilitating easier and more reliable appraisal of quality and relevance [9]. The Equator network [10], set up to actively promote their use, describes a reporting guideline as “A checklist, flow diagram, or structured text to guide authors in reporting a specific type of research, developed using explicit methodology” that “presents a clear list of reporting items that should appear in a paper and explains how the list was developed”. To improve the completeness of reporting of mobile health (mHealth) interventions, the World Health Organisation (WHO) mHealth Technical Evidence Review Group developed the mHealth evidence reporting and assessment (mERA) checklist [11]. Whilst aspects of this checklist are pertinent for RPM, no specific checklist for RPM applied to COVID-19 care currently exists.

The aims of this review of RPM for COVID-19 patients are twofold:

1. to facilitate comparison between RPM implementations by tabulating information and values under common domains.
2. to develop a reporting framework to enhance reporting consistency.

## Method

### Methods

A review of the literature for RPM for COVID-19 patients was conducted. Our research methodology included all required elements of the Preferred Reporting Items for Systematic Reviews and Meta-Analyses (PRISMA) checklist for systematic reviews [12] except for an assessment of the quality of the evidence as the articles considered were evaluated as informational reports of clinical implementations rather than as studies designed to answer a research question. Therefore, assessing the risk of bias within and across studies is not applicable to our study aims. Through the PRISMA review process we identified common definitions and then categorized the definitions based on the types of intervention characteristics considered in the definition.

### Search strategy

PICO elements were used to address eligibility criteria and search strategy. PICO represents an acronym for: (P) patient or problem, (I) intervention or exposure, (C) comparison intervention or exposure and (O) outcome of interest. In the present study, the relevant PICO elements were as follows: (P) patients being treated primarily for COVID-19 (I) Remote monitoring in the home; (C) not applicable; and (O) All relevant outcomes.

The Medline database was searched for articles published between 2020 to 2023, with a broad search strategy to identify all relevant studies published in English, where remote monitoring of patients during COVID-19 was the predominant issue. All articles were retrieved and indexed at the time of the literature search (February 2023). The keywords used in the search string were: “Remote Patient Monitoring”, “Home Monitoring” and “Virtual Monitoring”.

The full electronic search strategy for Medline was: (“Remote Patient Monitoring” OR “Home Monitoring” OR “Virtual Monitoring”) AND (“Covid-19”).

### Study selection and eligibility criteria

All abstracts derived from these searches were screened and analysed by the lead author based on the inclusion criteria: (1) peer-reviewed article, (2) in the English language, (3) with a focus on remote patient monitoring at home, and (4) dealt solely with COVID-19.

Full texts of all articles included at this step were reviewed by the lead author and discussed at research team meetings. Articles were excluded at this step if the study did not provide sufficient operational details of the intervention to allow for meaningful comparison, or they involved cases where COVID-19 was a complication of another illness, rather than the main illness being treated.

### Data synthesis and analysis

The lead author reviewed each article and extracted relevant data related to operational details and outcomes of each published manuscript.

Data were synthesised using thematic synthesis [13]. This type of synthesis follows three stages. Stages one and two involves coding text and developing descriptive themes whilst stage three is concerned with generating analytical themes. One of the selected studies providing detailed information on implementation and evaluation was selected to provide an initial coding template. Subsequent studies were evaluated and coded in light of this template, with additions and alterations made as appropriate.

In the second stage of analysis, similarities between codes were identified. Codes were grouped into ‘descriptive themes’ that described commonalities in the data across studies.

For stage 3, data within the descriptive themes was evaluated to determine consistencies, inconsistencies and variations between studies. The output from stage 3 was tabulated to allow for comparison between studies in terms of items reported and in terms of variations within reported items.

## Results

### Study selection

**Fig. 1:**
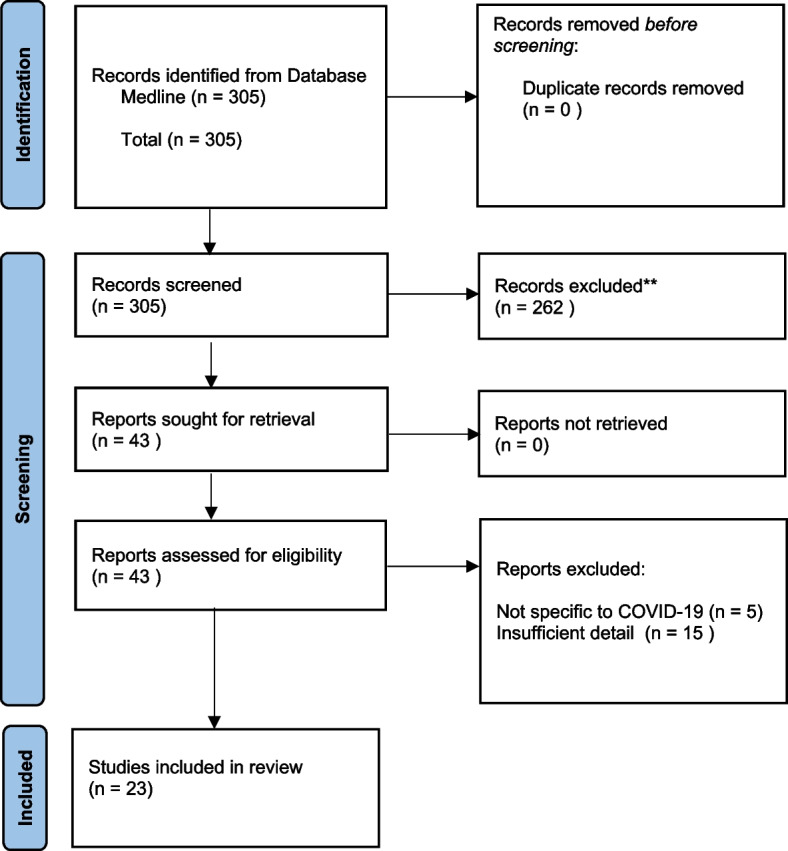
PRISMA flowchart

A total of 305 eligible abstracts were identified and screened and 43 met the inclusion criteria (Fig. 1). 23 studies were identified for review (Table 1), fourteen from the US, two from the UK and one each from Africa, Ireland, China, the Netherlands, Belgium, Australia and Italy.

**Table 1:** Overview of included studies

Ref	Reporting Period (Approx.) (2020)	Country	Number Enrolled	Patient Type
[14]	NA	US	83	Low to moderate risk ED patients
[15]	March to May 2020	US	2652	Confirmed / presumed COVID-19
[16]	NA	Ireland	26	COVID-19 positive with pulmonary infiltrates, without current need for supplemental oxygen
[17]	April, May 2020	US	112	Symptoms consistent with COVID-19
[18]	NA	Rwanda/Uganda	NA	COVID-19 cases and contacts in home isolation
[19]	NA	US	2000	Patients at risk of developing a serious case of COVID-19
[20]	January, February 2020	China	188	Home quarantined confirmed or suspected cases
[21]	April, May 2020	Netherlands	33	COVID-19 patients with clinical improving trend and oxygen therapy tapered down to a maximum of 3 L/min -f
[22]	April to June 2020	US	225	Patients with COVID-19 upon hospital discharge
[23]	May 2020	US	50	Low- and moderate risk COVID-19 with oxygen saturation of < 92% during the hospital stay
[24]	March to May 2020	US	2255	Patients with COVID-19 symptoms
[25]	April to June 2020	US	924	After testing positive to COVID-19 or after hospital discharge for COVID-19
[26]	May to June 2020	UK	192	Patients discharged from the ED with suspected COVID-19
[27]	April to June 2020	UK	279	Patients who were deemed likely to have COVID-19 pneumonia and were discharged
[28]	March to May 2020	US	154	Confirmed and suspected COVID-19
[29]	April May, 2020	Belgium	47	patients admitted to the pulmonology ward with a COVID-19 infection
[30]	April to June, 2020	US	80	patients with COVID-19, discharging who were considered to be high risk for clinical deterioration
[31]	April 13, 2020 through February 12, 2021	US	13,055	mild to moderate risk for hospitalization
[32]	April to August 2020	Australia	46	Moderate-risk and high-risk patients with Covid-19
[33]	March–October 2020	US	4,358	tested positive or under suspicion for COVID-19
[34]	January 2021 to November 2021	US	75	COVID-19 patients who required oxygen supplementation after hospital discharge
[35]	March 2020 to July 2021	Italy	200	a positive COVID-19 test with complicating co-conditions
[36]	March to December 2020	US	1234	moderate-to-high risk, positive for COVID-19

### Reported items

Sixteen reported items were identified and classified under four domains by the lead author: context, technology, process, and metrics as shown in Table 2 along with the percentage of reviewed studies reporting each item.

**Table 2:** Domains and items. Percentage of studies reporting each item is shown in brackets

Context	Technology	Process	Metrics
Dates (82%)	Provider (91%)	Markers (96%)	Patients Enrolled (96%)
Rationale (100%)	Communications Platform (100%)	Data Input Frequency (96%)	Alerts/Escalations (78%)
Patients (100%)	Patient Equipment (100%)	Thresholds (82%)	Patient Acceptance (43%)
Medical Team (91%)	Patient Training (48%)	Discharge (61%)	Patient Adherence (52%)

For all studies, information and values for the reported items were tabulated by domain (Additional file 1: appendices 1 to 4).

### Context domain

The individuals involved in the RPM implementation, both patients and health care provider staff are covered here, along with the rationale for patient enrollment and the dates on which monitoring occurred.

#### Dates [The specific time period for the reported RPM]

Four studies did not explicitly provide the dates covered [14, 16, 18, 19]. Most studies giving dates dealt with the early stages of the pandemic with 2 [31, 34] covering 2021.

#### Rationale [Reasons for implementation of RPM]

Two rationales for RPM were evident from these published studies: COVID-19 positive patients discharged from hospital to enable safe and early discharge with on-going RPM [14, 16, 21, 23, 26, 29, 30, 34]; and RPM for suspected or confirmed COVID-19 patients in the community to ensure only those patients who required hospital treatment were admitted [15, 17–20, 24, 28, 31, 33, 35, 36]. Two studies incorporated both [25, 32].

#### Patients [Patients included in RPM]

Patients were described in terms of confirmed or presumed COVID-19 positive [15, 22, 25, 26, 28, 29, 33], low to moderate risk [14, 23, 31], at risk of developing serious COVID-19 [19, 30, 32, 36], having symptoms of COVID-19 [17, 20, 24], had or have COVID pneumonia [16, 27] and have COVID-19 with complicating co-conditions [35]. The least ill patients were home quarantined suspected cases [18, 20] whilst the most complex were patients who, after hospital discharge, still required oxygen therapy at home [21, 34].

#### Remote monitoring medical team [Personnel involved in care of patients included in RPM]

Most medical teams included nurses and physicians [14–16, 19–21, 23–26, 29–31, 34, 36], one study also included a psychologist [21], another a neurosurgeon [17] and in one study [35] operations control centre staff were involved in monitoring. Medical trainees were also a feature, supervised by more senior staff [23, 25, 28]. One study noted that RPM allowed staff who were themselves quarantining to continue working from home by providing a service to remotely monitored patients [20].

### Technology domain

Technology played a major role in most implementations of RPM. However, some implementations did not use specialized technologies, merely phoning patients daily and asking about symptoms. This section covers the provision of technological approaches to RPM, the major messaging mode for transfer of information between patient and provider, the monitoring equipment provided to the patient, along with any training or instruction given to the patient in how to use the equipment.

#### Provider [Supplier of technology to enable RPM]

One site described sufficient in-house expertise to develop its own system for monitoring [13]. Some used proprietary systems from suppliers of medical monitoring systems [16, 17, 20–23, 25, 28, 33, 34] Others adapted systems that were in use prior to COVID-19 [14, 24, 32, 36] with the remainder using non-RPM specific systems such as the standard telephone system or packages [19, 26, 27, 29, 30] like Zoom or WeChat (A Chinese app comparable to WhatsApp). One African implementation used a text-based system from a non-profit orientated Canadian company called WelTel [18].

#### Communications platform [Type of communications used for patient to healthcare provider and vice versa]

One of the essential elements of any RPM system is the transfer of information from the patient to the provider at regular intervals. The method of transfer varies widely with mobile technologies often, but not always, involved. There is also the matter of automated transfer, without provider intervention or involving health care personnel. The simplest method is regular phone calls from care provider personnel to the patient with information transferred by phone. Two studies used this method [26, 27]. A further study [23] used this method initially but given the burden on staff found it was not sustainable and changed to a more automated system from a provider of RPM systems.

Three studies used standard text messaging. One [15] in the US sent a twice daily text message to initiate a text question and response exchange. Another from Africa [18] detailed a very similar system but with a daily semi-automated initial text. A further US study [33] used thrice daily text messages, interacting with proprietary software.

Email was used by two systems, one to send a link to a form at the start of monitoring which the patient was “required” to update daily [14], the other sent a link daily to a survey via email [19].

Use of mobile apps for data input and transfer was common, reported in 13 RPM implementations [16, 17, 21–25, 28, 30–32, 34, 35].

A web browser interface was used by 2 implementations [29, 36].

#### Patient equipment [Medical and other devices used as art of RPM]

Of the 23 implementations, 15 provided patients with a pulse oximeter on enrollment to enable patients to monitor their blood oxygen levels (SP02). Of these, eight also provided a thermometer [14, 19, 22, 28, 29, 31–33], two provided an oximeter only to high-risk patients [17, 26] with the remainder [16, 21, 23, 24, 27] providing an oximeter to all patients. In the case where no equipment was provided, three studies incorporated blood oxygen saturation (SPO2) and temperature measurements from patient owned oximeters and thermometers [17, 25, 26]. Four studies did not provide any equipment [15, 18, 25, 36] but one of these [25] used oximetry and temperature measurements where available. Two studies provided a high-tech bracelet device capable of measuring body temperature, heart rate, blood pressure, and oxygen saturation [30, 35].

Whilst some studies provided an oximeter and thermometer to high-risk patients, one study provided these to low-risk patients and provided high-risk patients with a cellular-enabled tablet telehealth system that monitored for blood pressure (BP), heart rate (HR), temperature, weight and SPO2 [19].

#### Patient training [Familiarization or training provided to patients specific to RPM]

Twelve studies did not mention any patient training or familiarization process [15, 16, 18–21, 28, 29, 32, 33, 35, 36]. For those that did, training took one of two forms: 1) provision of teaching materials such as leaflets or on-line videos and 2) personal outreach such as nurse contact. Five studies mention provision of teaching materials only [14, 24, 26, 27, 31], four used personal outreach only [23, 25, 30, 34] with two providing both [17, 22]. Only one report mentioned a technical support contact [17]. Whilst most studies concerned technical information such as how to use an oximeter or download an app, two [25, 26] provided non-technical COVID-19 information relating to home isolation and infection control.

### Process domain

Process describes various aspects of monitoring: what is monitored—known as markers, how often is it monitored (input frequency) and what thresholds are applied for escalation. It also covers discharge from the monitoring program, typically after a default period without complications, though some studies gave more specific criteria.

#### Markers [Physiologic and other indicators of health status monitored during RPM]

Patient data monitored, or markers, consists of physiologic data and self-reported symptoms.

Of the 16 studies that provided information on physiological markers, all included SpO2 [14, 16, 17, 19, 21–23, 25, 27–30, 32–35]. HR was reported by nine [14, 17, 19, 23, 30, 32–35], and temperature by 10 [17, 19, 21, 22, 25, 28, 29, 32–34]. Respiratory rate (RR) was included by four [17, 23, 30, 33].

All studies monitored symptoms, with 10 not indicating what these symptoms were [18, 19, 24, 26, 27, 30, 32–35]. Of the remaining 13, all monitored for dyspnea, four for cough [20, 22, 25, 36], diarrhea [17, 20, 25] and weakness [20, 22, 25], two for chest pain [17, 20], and two for vomiting [22, 26].

#### Data input frequency [How often marker information is sent to healthcare provider]

The frequency of data input by the patient varied across studies. 12 sites required once daily input [14, 17, 18, 20–22, 24–26, 28, 31, 36]; five twice daily [15, 19, 23, 32, 34]; 2 thrice daily [29, 33] and one 4 times daily [16]. 2 sites used a monitoring device that sent real-time data to a monitoring center [30, 35]. Most studies reported on some type of prompt sent to the patient via the transfer method when input is due. However, two sites requiring once daily input made no mention of prompts, simply stating that the patient was instructed [17] or required [20] to enter data once daily.

#### Thresholds for escalation [Levels of patient health status that initiate a cause for concern type alert to the healthcare provider]

Seven of the studies [15, 17, 20, 22, 24, 25, 28] reported that a patient can initiate an escalation themselves at any time via the RPM system.

New or worsening symptoms was specified by 11 studies [14, 15, 17, 19, 21, 22, 24, 25, 28, 29, 36].

For studies that monitored SpO2, a resting value of less than or equal to 94% [14, 16, 17, 27, 32, 35, 36], less than 92% [22], less than 90% [23], less than 88% [33], less than 85% [30] or when the difference in levels between resting and post exertion exceeded 5% [27] resulted in escalation.

One report gave a temperature of greater than 37.91 °C as a cause for escalation [22] whilst another [32] gave a value of 38 °C. Heart Rate (HR) threshold criteria for escalation, in beats per minute, were also reported: HR greater than 140 or less than 40 [30], HR greater than 130 or less than 50 [32], HR greater than 105 [14], HR greater than 100 or less than 60 [35], HR greater than 100 [17], HR greater than 115 at rest or greater than 125 twenty seconds post exertion or a difference greater than 10 pre – post exertion [23].

Escalation values for RR, in breaths per minute, were: greater than 30 or less than 8 [30], greater than 20, [17], greater than 22 at rest or greater than 30 at 20 s post exertion or a difference greater than 8 pre – post exertion [23].

#### Discharge [Conditions under which a patient is discharged from RPM]

Fourteen days was the typical default timescale for leaving the monitoring programme. One study specified 8 days [30], with 2 specifying 10 days [35, 36]. Of the studies that gave specific clinical criteria to enable discharge, two required oxygen saturation levels to be greater than 96% [14, 23] for 3 days with one requiring normal oxygen saturation level for 3 days [27]. The normal level was not specified by the studies but is usually considered to be 95% to 100% [37]. One of these [14] also required a heart rate of less than 100 bpm and a temperature of less than 37.96**°** C. One study allowed patients to optionally extend the monitoring period from 14 to 21 days [22]. One study [29] discharged when the patient felt better and re-engaged in their daily activities.

### Metrics domain

Numbers can allow us to appreciate the scale of monitoring more fully for each report and provide comparative information on how many patients required escalation. In this section, we provide the number of patients enrolled in each implementation and the numbers of those who were escalated. Also included here are metrics regarding patient acceptance of RPM and adherence to the data inputting requirements.

#### RPM enrollment [Number of patients enrolled in the RPM implementation]

All but one study reported numbers for patients enrolled in RPM. Seven involved between 1,234 and 13,055 patients [15, 19, 24, 25, 31, 33, 36]; seven involved between 112 and 295 patients [17, 20, 22, 26–28, 35]; while eight involved between 26 and 83 patients [14, 16, 21, 23, 29, 30, 32, 34]

#### Escalation [Numbers of patients in RPM program requiring intervention beyond baseline care]

Escalation involved an admittance or readmittance to hospital, or merely a short interaction via phone or video with a health care worker. Only a minority of patients typically escalate. For one large programme with over 2000 patients enrolled, 83% were managed without escalating to human care [15]. The largest study in this review, with 13,055 enrolled, stated that 10% of patients were escalated to hospital care [31]. 10% was also given as the number escalated to ED in a study involving 1,234 patients [36]. Another study, with just under 1000 enrolled, reported that about 10% of patients presented with symptoms requiring escalation to a virtual provider, and 2% required admission to hospital [25]. For one of the smaller implementations with 26 enrolled [16], the 26 patients generated 51 alerts, which in turn generated 5 reassessments leading to readmission of 4 patients. However, a study with 83 participants [14] stated that 60 patients triggered an automated flag at least once, 39 patients were escalated to a telehealth consult and 17 patients were referred to the ED.

#### Patient acceptance of RPM [How well the RPM program was accepted by patients]

Nine studies reported patient feedback on RPM. All reported high acceptance. Two reported a high net promoter scores of 80 [15] and 71.5 [33]. The net promoter score is a single metric that quantifies the response to a single direct survey question: How likely are you to recommend this service? [38]. 91% of patients provided feedback in one study using a satisfaction questionnaire based on Consumer Quality Index in General Practice [21], with 97% of those finding the system user friendly. For five studies [23, 27, 31, 32, 34] the proportion of respondents to a survey that would recommend the service to a friend was 94%, 99.5%, 94%, 88% and 100% respectively.

#### Patient adherence [Compliance with patient requirements to provide health status data to healthcare provider]

Patients were requested and usually prompted to input their data to the system on a regular basis. How well they complied, or patient adherence, has been indicated by 12 of the studies to varying extents. Adherence varied. One study involving hospital discharge [16] that prompted patients 4 times daily for input showed a median daily input of 3.9 for those that did not require readmission and 5.7 for those readmitted, indicating high adherence. Another study involving hospital discharge requiring daily input indicated that patients were monitored for an average of 21.8 days and completed an average of 14.5 daily survey responses suggesting a somewhat lower adherence [14]. A study sending twice daily check-in prompts saw a 59.7% response to both, 27.5% to one and 12.8% to neither [15]. Another noticed a drop off in compliance as RPM progressed, with 91% performing at least one daily measurement and 68% all three between days one and four, but stating that compliance declined significantly after this [29]. The average number of completed responses to a three times daily text prompt, was high at 87.2% for another study [33].

### Reporting consistency

The types of information reported on varied across studies. Four did not provide any date information [14, 16, 18, 19]; 12 did not provide details of patient training [15, 16, 18–21, 28, 29, 32, 33, 35, 36]; one did not provide information on markers or input frequency [26]; four did not provide details of escalation thresholds [18, 20, 31, 34],; eight did not indicate discharge conditions [17, 19–21, 31–34]; one did not indicate number of patients enrolled [18]; five did not indicate number of escalations [18, 19, 30, 33, 35]; 14 gave no indication of patient acceptability [14, 16–20, 22, 24–26, 28–30, 35] and eleven gave no indication of patient adherence [10, 18–20, 26–28, 30, 34–36]. Just one mentioned staff training [30] and none mentioned staff acceptance. This is not a criticism of any study but strongly indicates a need for greater consistency in reporting of RPM implementations to support learning and meaningful comparison.

### Framework

Based on the domains and items outlined above, and with the addition of health staff acceptance and training mirroring patient considerations, we propose a framework for reporting of RPM for COVID-19 patients as shown in Table 3.

**Table 3:** Framework for reporting on RPM studies for COVID-19 patients

DOMAIN	Item	Item no	Notes
CONTEXT	Dates	1	Clearly state the implementation dates covered by the study
	Rationale	2	State the specific purpose and context of the implementation, e.g., step-down, preserve bed capacity, increase personnel efficiency, prevent iatric outcomes
	Patients	3	State type of patient catered for by implementation, illness severity, suspected or confirmed cases etc
	Medical team	4	Detail range of medical personnel involved in the implementation: roles, medical specialties, seniority, dedicated or shared etc
TECHNOLOGY	Technology provider	5	Provide details of the technology provider: inhouse build, adoption of system already in place, commercial provider along with name and head-office location
	Communication mode	6	Detail the provider patient mode of communication e.g., text, web form, phone call, smartphone app
	Patient equipment	7	Describe the type and make of patient equipment utilized and detail how it is provided e.g. user provided, provided by healthcare system and how it is delivered and returned
	Patient training	8	Describe what patient training is provided regarding equipment, procedures and self-care and how this training is conducted
	Staff training	9	Describe what staff training is provided regarding equipment, procedures and communication with patients and how this training is conducted
PROCESS	Markers	10	Detail what is monitored and how, specifically physiologic markers and self-reported symptoms
	Data Input Frequency	11	Detail how often patient data is input to the RPM system, whether done automatically or manually by the patient and describe the frequency and mode of any prompts for input provided to the patient by the system. Outline the procedure followed when input is not received
	Thresholds for Escalation	12	Clearly present the escalation procedure. Detail marker thresholds that trigger a cause for concern alert and how such an alert is sent and received. Detail the number and types of escalation stages, e.g., initial screening by paramedic followed, if deemed apt, by further escalation to physician
	Discharge	13	State the conditions under which a patient is discharged from RPM e.g., after certain duration, marker thresholds, clinical judgement
METRICS	RPM Enrollment	14	Clearly state the number of patients monitored over the course of the reported implementation along with subgroup breakdowns
	Escalation	15	Provide numbers for cause for concern alerts, and escalations by level
	Patient acceptance of RPM	16	Report on patient acceptance using net promoter score or other suitable instrument
	Staff acceptance of RPM	17	Provide some indication of staff acceptance using suitable scale
	Patient Adherence	18	Report on the frequency of patient data input compared to the prescribed frequency

## Discussion

This study explored descriptive reports of RPM programmes to illustrate the variety of, and provide details on, such programmes in a manner that facilitates rapid familiarisation. It is not the intention here to determine whether one mode of implementation is better than another. However, the wide variety seen in the studies is relevant, suggesting that RPM for COVID-19 is still at an early stage.

The RPM implementations described here were put in place in the early stages of the pandemic, so perhaps it is not surprising that there is a high level of variation. Even within individual studies, changes occurred as lessons were learnt over time. One programme [23] changed from monitoring patients by phone to a system of automated monitoring to reduce demands on personnel. Another study [15], having increased the number of symptoms monitored, reduced back to the original number due to a resulting large increase in unwarranted escalation calls.

The equipment provided to patients varied. The use of pulse oximetry in RPM for COVID-19 is well demonstrated here and is itself a separate subject of research [39]. However, not all health systems will be able to provide the required devices. It is interesting, therefore, to note that several studies from health systems in advanced and developing countries with varying degrees of resource availability used only self-reported symptom data. A study in China [20] developed a set of quarantine management scales concluding that these worked well in identifying patients with disease progression. Somewhat contrary to this, a UK study bemoaned the fact that pulse oximeters were not used more, as their experience was that pulse oximetry enhanced telephone assessment of patients. Wide variation is also seen in the infrastructure used for RPM. Infrastructure ranged from the standard telephone system to complex proprietary systems from companies specialising in remote patient monitoring. Markers also varied, ranging from a simple report of symptoms to combined physiological measures such as HR, SPO2 and RR. Thresholds for escalation ranged from a self-report on worsening symptoms to algorithms using personalised thresholds.

It remains to be seen how much increased complexity and sophistication might enhance patient outcomes.

Information reported on also varied, again perhaps due to the studies being conducted at the early stages of the pandemic. Regarding the importance of reporting consistency in the area of RPM, we note once again the comments made in a systematic review on RPM for COVID-19. The authors stated that it was difficult to carry out an analysis of the impact of RPM across all examples in the review because not all articles reported data on the same outcomes. Substantive conclusions regarding patient safety and the identification of early deterioration could not be reached due to lack of standardised reporting and missing data [8].

Two reporting guidelines already developed have relevance to RPM, the CONSORT E-health guidelines [40] and the mERA checklist [11]. The CONSORT guidelines seek to improve and standardize evaluation reports of web-based and mobile health interventions, whilst the mERA checklist, developed by the WHO mHealth Technical Evidence Review Group, seeks to standardise the quality of mHealth evidence reporting, and so indirectly improve the quality of mHealth evidence. However, these guidelines are broad in scope, designed to cover a wide range of mobile health and e-health studies. To our knowledge this is the first review to propose a key set of reporting items for COVID-19 RPM. Use of the framework will enhance consistency and aid analysis across studies.

### Limitations

Whilst the search for information conducted for this study was thorough; it was limited to studies reporting sufficient information on implementations to provide meaningful comparisons and did not consider health outcomes. As such, certain aspects of RPM may be omitted. This review largely considers aspects of clinical implementation and does not include non-clinical matters such as purchase and storage of monitoring equipment or cost–benefit analysis.

### Future research

We believe that the information presented here will allow for rapid familiarisation for those seeking an overview of RPM for COVID-19 and, by using the suggested framework, enhance the consideration and reporting of planned and existing RPM implementations for COVID-19. The work here also suggests areas for future research.

The difference in implementations suggests the need for further research to determine if and under what conditions a simple implementation of RPM involving a phone call or text to report on symptoms is adequate, and under what conditions increased benefits may ensue with increasing complexity. This may be particularly important for developing countries as they tackle the pandemic. The details outlined in this study can help inform what needs to be included in such studies.

Future research may also extend the framework to include more non-clinical aspects of RPM, such as reporting on cost–benefit analysis and technical integration.

The reporting framework presented here may be seen as an initial step towards a more robust set of reporting guidelines with future research advancing the framework, using a Delphi methodology or similar, to inform standardised reporting guidelines for work of this nature.

## Conclusions

Variations in reported items were found. Pending the establishment of a robust set of reporting guidelines, we propose a reporting framework consisting of eighteen reporting items under the following four domains: Context, Technology, Process and Metrics. We believe that the framework presented here, used as a key set of reporting items, will enhance the consideration and reporting of RPM studies for COVID-19 and allow for enhanced comparison and analysis across studies.

### Supplementary Information

**Additional file 1. Appendix 1.** Context Domain. **Appendix 2. **Technology Domain. **Appendix 3. **Metrics Domain. **Appendix 4.** Process Domain.

## Data Availability

All data generated or analysed during this study are included in this published article [and its supplementary information files].

## Abbreviations

BP: Blood pressure
FDA: Food and Drugs Administration
HR: Heart Rate
mERA: MHealth Evidence Reporting and Assessment
mHealth: Mobile Health
RPM: Remote Patient Monitoring
RR: Respiratory Rate
SPO2: Blood oxygen level
WHO: World Health Organisation
