# Supplementary material for: Remote patient monitoring for COVID-19 patients: comparisons and framework for reporting
Source: BMC Health Serv Res. 2023 Aug 3;23:826. doi: 10.1186/s12913-023-09526-0 (PMC10401771; doi:10.1186/s12913-023-09526-0)
Supplement: Supplementary file 1 — Additional file 1. Appendix 1. Context Domain. Appendix 2. Technology Domain. Appendix 3. Metrics Domain. Appendix 4. Process Domain. [file 12913_2023_9526_MOESM1_ESM.docx]

**Appendix 1 : Context Domain**  NA: Not apparent

|  | **CONTEXT** | | | |
| --- | --- | --- | --- | --- |
| **Ref** | **Reporting Period (Approx) (2020)** | **Rationale** | **Patients** | **Medical Team** |
| 14 | NA | Discharge | low to moderate risk ed patients | Emergency medical technicians and emergency physicians |
| 15 | March to May | Triage | confirmed / presumed COVID-19 | Nurses, physicians, and advanced medical practitioners. |
| 16 | NA | Discharge | COVID-19 positive with pulmonary infiltrates, without current need for supplemental oxygen | respiratory physicians and nurse specialists. |
| 17 | April, May | Triage | symptoms consistent with COVID-19 | practitioners, physiatrists, and neurosurgeons |
| 18 | NA | Triage | COVID-19 cases and contacts in home isolation | health officials |
| 19 | NA | Triage | patients at risk of developing a serious case of COVID-19 | nursing team |
| 20 | January, February | Triage | Home quarantined confirmed or suspected cases | remote monitoring nursing teams. |
| 21 | April, May | Discharge | COVID-19 patients with clinical improving trend and oxygen therapy tapered down to a maximum of 3 L/min -f | physicians, nurses, psychologist |
| 22 | April to June | Discharge | patients with COVID-19 upon hospital discharge | medical residents supervised by pulmonologist |
| 23 | May | Discharge | low- and moderate risk COVID-19 with oxygen saturation of <92% during the hospital stay. | team of triage nurses with physician backup |
| 24 | March to May | Triage | patients with COVID-19 symptoms | nurses with escalation to physicians |
| 25 | April to June | Both | after testing positive to COVID-19 or after hospital discharge for COVID19. | medical students, residents, supervising physicians, nurse practitioner and np students |
| 26 | May to June | Discharge | patients discharged from the ED with suspected COVID-19. | nurses and clinicians |
| 27 | April to June | Discharge | patients who were deemed likely to have COVID pneumonia and were discharged | led by an infectious disease specialist |
| 28 | March to May | Triage | confirmed and suspected COVID-19 | Physician Associate students supported by Emergency Department Associate Specialist doctors. |
| 29 | 8 April and 9 May, 2020 | Discharge | patients admitted to the pulmonology ward with a COVID-19 infection | specialised telemonitoring nurses |
| 30 | April 15, 2020, to June 6, 2020 | Discharge | patients with COVID-19, discharging who were considered to be high risk for clinical deterioration | nurse and/or an attending physician |
| 31 | April 13, 2020 through February 12, 2021 | Triage | mild to moderate risk for hospitalization | nurses and physicians |
| 32 | April to August 2020 | Both | Moderate-risk and high-risk patients with Covid-19 | NA |
| 33 | March–October 2020 | Triage | tested positive or under suspicion for COVID-19 | NA |
| 34 | January 7, 2021 to November 11, 2021 | Discharge | COVID-19 patients who required oxygen supplementation after hospital discharge | registered, licensed vocational, and licensed practical nurses |
| 35 | March 2020 to July 2021 | Triage | a positive COVID-19 test with complicating co-conditions | Operations Control Centre staff, nurses, physicians |
| 36 | March 30 and the end of December 2020 | Triage | moderate-to-high risk, positive for COVID-19 | nurses |

**Appendix 2 : Technology Domain NA: Not apparent**

|  | **TECHNOLOGY** | | | |
| --- | --- | --- | --- | --- |
| **Ref** | **Provider / Infrastructure** | **Communications Platform** | **Patient Equipment** | **Training** |
| 14 | In house-adapted existing software platforms | email | pulse oximeter and thermometer | Informational, educational and consent documents with videos and electronic and physical teaching materials |
| 15 | adapted systems already available | text messages | none | NA |
| 16 | Prorietary RPM system from patientMpower Ltd, Dublin, Ireland. | app | pulse oximeter | NA |
| 17 | Proprietary non-RPM: MyCap, Zoom | app with video meetings | pulse oximeter | Verbal instruction re apps. Virtual guide and contact information for technical support. |
| 18 | Proprietary non-profit, non-RPM: WelTel virtual care system, WelTel Health, 2020. | text message | none | NA |
| 19 | Uses videoconferences and phone calls. Patient inputs via online patient portal. | patient portal account | thermometer and pulse and tablet telehealth | NA |
| 20 | Proprietary non-RPM: Chinese social media smartphone app, WeChat | app | none | NA |
| 21 | Proprietary RPM system from Luscii, Amsterdam, the Netherlands | app. | pulse oximeter | NA |
| 22 | Proprietary RPM: MyChart Care Companion, | app | pulse oximeter and thermometer | instructional packet provided. patients contacted by nurse to provide assistance |
| 23 | Proprietary RPM system from Around the Clock Alert | app | a pulse oximeter | Vendor assisted patients with app. A follow-up call tested the equipment and acclimatized the patient to the program. |
| 24 | Proprietary RPM (GetWell Loop) already used in house adapted for COVID-19 | app | pulse oximeter | Patients receive an email on how to activate and begin the COVID-19 program |
| 25 | Proprietary RPM MyChart Care Companion app | telephone and app | pulse oximeter and thermometer | patients receive a call with instructions on home isolation and COVID-19 and to screen for concerns about social support and home safety, and to invite them to engage with the MyChart Care Companion app |
| 26 | Standard telephone system | telephone | pulse oximeter | All patients were given an information leaflet with advice on infection control, self-monitoring, indications to reattend for assessment, and a contact phone number for clinical advice in hours |
| 27 | Telephone system | telephone | pulse oximeter | patient leaflet which included the contact details for the virtual ward link to a website with video information of how to use the oxygen saturations monitor |
| 28 | The in-house tool and proprietary RPM MyChart app | app | pulse oximeters and thermometer | NA |
| 29 | secure hospital server | web browser, telephone or text | automated blood pressure monitor, a portable fingertip saturation probe and a thermometer | NA |
| 30 | secure cloud server | App | Masimo Radius PPG bracelet | The VHC team placed a call to the patient’s house the evening of enrollment to ensure a smooth transition of care |
| 31 | NA | App or via computer | a pulse oximeter, thermometer | Handouts and patient videos |
| 32 | RedCAP and pre-existing hospital information technology infrastructure | app / sms | pulse oximeter, digital thermometer | NA |
| 33 | Twistle, a HIPAA compliant patient engagement platform operated by Health Catalyst | text messages | thermometers and pulse oximeters | NA |
| 34 | RPM platform (Vivify Health) and a nurse-monitoring service (Global Medical Response) | app | disposable vital signs monitoring equipment | registered nurses provided patients with education on use of the RPM application and equipment |
| 35 | NA | app | Masimo’s Radius Tº) consisted of a wireless wearable device which continuously measures and tracks a patient’s body temperature, heart rate, blood pressure, and oxygen saturation | NA |
| 36 | adapted already existing infrastructure and RedCAP | Online patient portal | None provided | NA |

**Appendix 3 : Metrics Domain** NA: Not apparent

| Ref | **METRICS** | | | |
| --- | --- | --- | --- | --- |
|  | **Enrolled** | **Alerts/Escalated** | **Patient acceptance** | **Adherence** |
| 14 | 83 | 60 patients triggered an automated flag, 39 escalated to a telehealth consult and 17 referred to the ED. | NA | Patients completed an average of 14.5 (3–57, median 13) daily survey responses. |
| 15 | 2652 | 396 patients escalated to clinical care with 83 patients recommended to go to the ED. | a net promoter score of 80 from the ratings of 554 patients (24% of those participating). | As a daily average: 59.7% responded to both check-ins, 27.5% to 1 and 12.8% to neither |
| 16 | 26 | 51 alerts generated 5 reassessments leading to readmission of 4 patients. | NA | median daily inputs: 3.9 for no readmission, 5.7 in those readmitted. |
| 17 | 112 | 20 patients were triaged and 6 of these were referred to ED. | NA | 83% downloaded app, 17% data collected via zoom or phone call with physician |
| 18 | NA | NA | NA | NA |
| 19 | 2000 | NA | NA | NA |
| 20 | 188 | 6 patients progressed to critical care | NA | NA |
| 21 | 33 | Reassessment at the hospital was indicated in six (18%) | Satisfaction questionnaire was completed by 30 (91%) patients. Ninety-seven percent rated home telemonitoring as user friendly | NA |
| 22 | 225 | 72 patients alerted with 11 to ED and 3 to hospital | NA | 210 patients completed at least one questionnaire; 2,161 total questionnaires completed |
| 23 | 50 | 13 patients generated 29 alerts leading to 3 ED referrals and 1 readmission | 46% provided feedback 94% saying they would recommend to a friend | "High compliance with numerous daily vital submissions" |
| 24 | 2255 | patients generated 2303 alerts leading to 91 referred to ED and 13 admitted to hospital. | NA | There have been 10 770 patient check-ins. Of patients who activated their accounts, 94% checked in at least once. |
| 25 | 924 | 10% of patients require escalation to a virtualist provider, 2% require admission to hospital. | NA | Current engagement with the MyCare Companion app is at 32%. |
| 26 | 192 | 32 reattendances | NA | NA |
| 27 | 279 | 31 required reassessments in hospital | 66% provided feedback with 99.5% being likely or very likely to recommend to a friend | NA |
| 28 | 154 | 22 referred for physician review with 4 referred to ED | NA | NA |
| 29 | 47 | A total of 1259 measurements were registered, of which 5% triggered a phone call by a specialised nurse and 2% triggered a phone call by a physician | NA | From day one to day four, 91% of the patients performed at least one daily measurement, and 68% performed all three. After day 5, compliance declined significantly |
| 30 | 80 | NA | NA | NA |
| 31 | 13,055 | "Patients triggered an average of 3.39 alerts during daytime hours and 1.05 alerts during afterhours throughout their length of stay on the program, 10% of | "(56%) responded to a patient satisfaction survey, with 94% of respondents stating they would recommend the Home | Patient adherence to the administered care plan was also high at 94% |
| 32 | 46 | enrolled patients have been escalated to hospital care f" | Monitoring program to a family member or frien" | median number of self-reported observations submitted by patients through the HMP was 16 |
| 33 | 4,358 | 10 / 46 generated alert | 14 of 16 (88%) surveyed would recommend | Engagement with the interface, defined as the average number of completed responses to the three times daily text prompts, was high (87.2 ± 23.3%) |
| 34 | 75 | NA | overall net promoter score was 71.5 | NA |
| 35 | 200 | "1556 biometric alerts that were triggered. Fewer than 1% (8/1556) of biometric alerts required | (100%) of respondents indicated they would recommend the remote monitoring program to others | NA |
| 36 | 1234 | escalation to the on-call clinician" | NA | NA |

**Appendix 4 : Process Domain** NA: Not apparent

|  | **PROCESS** | | | |
| --- | --- | --- | --- | --- |
|  | **Markers** | **Frequency of prompt / Input** | **Thresholds** | **Discharge** |
| 14 | SPO2 , resting HR, shortness of breath | once daily | SPO2 88-93%, resting HR>105, worsening or new shortness of breath | 10 days after symptoms started or monitoring initiation with 3 days symptom improvement or no symptoms. Last 3 days: O2>96%, HR<100 (unless elevated at baseline), temp<100.4, |
| 15 | breathing difficulty self-report | twice daily | Patients who reported dyspnea in response to the twice daily check-ins, or who texted “worse” at any time, | 14 days after enrolment with optional 7 days extra |
| 16 | SPO2, breathlessness | 4 times daily | oxygen saturation <=94% | after 14 days |
| 17 | symptoms and physiological data | daily | clinical deterioration, new onset dyspnea and chest pain, SPO2< 94%, HR>100, >20breaths/min, | NA |
| 18 | new symptoms or issues | daily | NA | 14 days |
| 19 | blood pressure, heart rate, temperature, weight, and oxygen saturation and vital signs and symptoms. | twice daily | vital signs or symptoms change beyond predefined thresholds. | NA |
| 20 | Fever, Mental state, Muscle soreness, Cough, Dyspnea, Lack of strength , Diarrhea , Chest tightness | daily | NA | NA |
| 21 | oxygen saturation, temperature, respiratory symptoms | daily | personalized thresholds | NA |
| 22 | shortness of breath, cough, appetite, weakness, vomiting, O2 saturation, and temperature | daily | worsening symptoms, O2 < 92%, temperature > 100.3F; assigned monitoring task had not been completed in 24 hours | 14 days with option to extend to 21 |
| 23 | SaO2, HR, RR | twice daily | SaO2 < 90%, or decrease of >5% exertion +20-s rest HR >115 at rest, or HR >125 exertion +20-s rest, or increase of HR >10 between rest and exertion +20-s rest RR RR >22, or RR >30 exertion +20 s, or increase in RR >8 between rest and exertion +20-s rest | 14 days and oxygen saturation > 96% for three consecutive days. |
| 24 | symptoms | daily | concerning answers to symptom questions - patient initiate | 18 days initially, adjusted to 16 days, |
| 25 | cough, dyspnea, weakness, vomiting, diarrhea, appetite, SP02, temperature | daily | new or worsening symptoms | 7 days after discharge or 14 days from symptom onset |
| 26 | NA | NA | clinically indicated | NA |
| 27 | SPO2 and symptoms | daily | Saturations <=94% at rest OR <=90% in a COPD patient with chronic hypoxia) or a >5% drop following exertion. | normal oxygen saturation for five days and symptoms improving |
| 28 | dyspnea, SPo2, temperature | daily | symptoms worsen | 14 days |
| 29 | BP, SPO2, Temperature, dyspnoea, fever, general condition | three times daily | "deterioration of vital signs or symptoms was noted or the patient wrote remarks or questions | when patient felt better and re-engaged in their daily activities |
| 30 | respiratory rate, heart rate, and pulse oximetry | realtime data transmission from the app | in the free text box" | 8-day monitoring period |
| 31 | cough, shortness of breath, confusion, and other indicator | once daily | RR<8 or >30 for 10 mins, PR <40 or > 140 for 10 mins, SPO2 < 85% for 10 mins | NA |
| 32 | symptoms, pulse rate, temperature and oxygen saturation | twice daily automated prompts | NA | NA |
| 33 | oxygen saturation, respiratory rate, heart rate, temperature and symptoms. | thrice daily | HR<50 or >130, SPO2<95%, Temp>38c | NA |
| 34 | BP, O2, HR, Steps,Temp, Symptom report | twice daily | oxygen saturation of 88% or less requiring immediate response from the clinical team | NA |
| 35 | oxygen saturation, blood pressure, heart rate, and symptoms | Realtime Biometric | NA | not exceeding 10 days from the outcome of their positive COVID-19 test, coincides with the end of the device battery |
| 36 | shortness of breath, cough | daily | SpO2 <94%, and heat rate <60 and >100 beats at rest, and BP alteration | 10–20 days |
